# Implications of salep collection for the conservation of the Elder-flowered orchid (*Dactylorhiza sambucina*) in Epirus, Greece

**DOI:** 10.1186/s40709-019-0110-1

**Published:** 2019-12-30

**Authors:** Martha Charitonidou, Kalliopi Stara, Konstantinos Kougioumoutzis, John M. Halley

**Affiliations:** 1 Laboratory of Ecology, Department of Biological Applications & Technology, University of Ioannina, 45110 Ioannina, Greece; 2 Department of Ecology and Systematics, Faculty of Biology, National and Kapodistrian University of Athens, 15784 Athens, Greece

**Keywords:** Orchids, Salep, Non Timber Forest Products (NTFP), Wild products, Illegal trade, Conservation value, Northern Greece, Northern Pindos National Park

## Abstract

**Background:**

In Epirus, Greece, orchids have been traditionally harvested for the production of salep, a beverage made from their tubers. Over-collection of orchids for salep is believed to be a growing threat to wild species, yet very little research has concentrated on orchid populations in the wild. Here, we studied the impact of salep collection on population demographic parameters and uniformity of distribution patterns of the Elder-flowered orchid, *Dactylorhiza sambucina*, the most commonly collected orchid in northern Greece.

**Methods:**

We carried out fieldwork in four meadows where salep harvesting occurs, and conducted interviews in villages close to these sites. Fieldwork focused on the demographic parameters of orchid populations and on the characteristics of their habitat (natural-anthropogenic). We also measured population size and distribution, extent and multi-scale density, comparing distributions to Poisson and fractal models.

**Results:**

According to interviews, salep collection by the local community has decreased, contrary to collection by people outside the community, which is increasing. Interviewees did not believe that orchid abundance was higher in the past; they claim that it can be very variable. None of the participants seemed aware of the legislation to conserve orchids. Demographic parameters did not seem to be strongly dependent on whether it was a harvested and non-harvested sites and population density was greatest in the site of highest collection pressure.

**Conclusions:**

Our findings show that salep collection is still ongoing in Epirus. Our interview results and our population study indicate that current levels of collection are not significantly affecting the abundance of the Elder-flowered orchid in Epirus subalpine meadows. However, the expanding commercial collection could reach levels that threaten the species. There is a need for a longer-term monitoring of these orchid populations, and a more effective modeling of the species’ response to different harvesting pressures.

## Background

The Orchidaceae, one of the oldest vascular plant families (76–112 My old) [1–3], have an almost global distribution, yet they are absent from the deserts and the poles [4, 5]. They are also the most diverse vascular plant family comprising ca. 736 genera and ca. 28,000 species [6, 7]. Since their first mention in Chinese medical texts around 2800 BC [8], orchids have always been in the spotlight of plant enthusiasts and scientists. The allure of their beauty, their striking appearance and their “endless diversity of structure” [9] has excited a curiosity that has sometimes been detrimental to orchid conservation. From at least the nineteenth century, orchids have been over-collected for scientific, ornamental and medicinal purposes [10–13], which has arguably brought many species to the edge of extinction [14]. Regarding this, several orchid species have been assessed by IUCN, with the threat status for the ones that are directly threatened varying from Vulnerable to Critically Endangered [15]. In terms of legislative actions, many orchid species are included in local (the Greek Presidential Decree 67/1981), regional (Annexes A and B of the Council Directive 92/43/EEC in the European Union) and global (Appendices I and II of the Convention on International Trade of Endangered Species of Fauna and Flora-CITES) protection/conservation laws, that prohibit their collection and harvest, as well as any commercial use [16].

Since ancient times [as mentioned in works by Theophrastus (ca. 300 B.C.), and later on, Dioscorides (ca. 60 A.D.)], orchids have been collected in the Eastern Mediterranean for their putative healing properties. Orchid harvesting continued during the Ottoman Rule (mid fifteenth to early twentieth century), while recently, the pace of orchid harvesting has been increasing again [17], because of the uprising market demand for *salep* (Greek: *salepi*), a flour made from dried orchid tubers [18, 19]. The etymology of the word *sālep* derives from Arabic *(ḵuṣa*-*’ṯ*-*) ṯa‘lab*, the name for an orchid that literally means ‘fox’s testicles’ [20]. Salep is popular primarily in Turkey, but also in Greece, Iran, Iraq and Albania [21, 22]. It is mainly sold by street vendors during winter, as a remedial hot beverage for cold and cough in the aforementioned countries [23]. It has also a long tradition in gourmand and culinary products as key ingredient in the famous *dondurma* (Kahramanmaras) ice cream in Turkey [24] and *kaimaki* ice cream in Greece [17]. However, salep overharvesting is a serious threat increasing the extinction risk of orchid species in the region [25, 26]. Orchids that are collected for salep, belong to genera that bear ovoid bulbs or palmate tubers. In general, salep is made from at least 35 different species of orchids [27], with *Anacamptis, Orchis* and *Dactylorhiza* species being the most common “salep orchids” [18].

Greece is highly diverse in orchids, with Epirus (northwest Greece) being among the most species-rich areas, hosting ca. 70 taxa [28, 29]. The most commonly harvested species for salep in Greece are *Anacamptis morio*, *Dactylorhiza sambucina, D. saccifera* and *Orchis mascula* [26, 30]. Other orchids, mainly from the genera *Anacamptis* and *Orchis* are also mentioned from older references [31–33]. A large fraction of the salep collected in Greece and neighbouring countries, finds its way to the international market mainly through the Netherlands, Northern Cyprus [34], and especially Germany, which is by far the largest trader in medicinal and aromatic plants [35]. The current high demand has also resulted in the increasing use of substitutes, such as rice powder and carboxymethyl-cellulose (CMC) to meet the market needs [34]. However, there has been a revived demand for authentic salep, along with other forest food plants and “Non-Timber Forest Products” (NTFP) in general, as part of modern people’s desire to be reconnected with Nature, with traditional culture and with their own locality [36]. Ironically, this yearning for reconnection with Nature may actually drive local extirpation for many vulnerable species, including orchids [16, 22, 37] because of its potential magnitude. For example, in Europe, the official value of the market in plant and mushroom products alone was estimated at €1.68 billion [38]. The unregulated and undocumented orchid harvest and trade relating to salep is expected to be very large and to put pressure on populations in the wild. To that end, research into trade dynamics and the impacts of harvest are very important.

Although several relevant studies have been carried out dealing with the ecology, genetic diversity and conservation of these orchids [39–42], less work has been done on orchids specifically collected for salep [26, 37]. Existing studies outline the threat to orchid populations, but there has been very little examination of populations themselves. Since population is such an important concept in conservation, we need an integrated approach that will examine the impact of salep collection on orchid populations [43]. Thus, our aim is to investigate the impact of salep harvesting on population demographic parameters and distribution patterns of *Dactylorhiza sambucina* in Epirus, interviewing local collectors and considering the background cultural history of orchid harvesting.

## Results

### Results from interviews

Salep collection is not an easy task; there are only a few people in every village we visited, who know the plants and have inherited the practice as a family tradition. From the six commercial collectors we interviewed, five still collect salep, as a supplementary occupation and the quantities mentioned are 10–100 kgr of dry tubers per collector or collectors (e.g. husband and wife) per season. Collection is also an attractive activity for economic migrants who work in Greece as shepherds and supplement their low salaries by collecting from the wild plants of economic interest including salep. Prices depend on networking, negotiation and the collector’s position in the product value chain and they can vary from 10 to 80 euros per kilo of dry tubers, while ground salep price in the market rises at 120–180 euros per kilo. These people collect mainly *Dactylorhiza sambucina,* though they are also familiar with other orchid species, based on the results of our interviews. They distinguish the species by the different shape of its tubers (oval or palmate) and by the habitat they find it (forest edges, wet places, summer pastures). They collect all species mainly from early June to mid-July, when they are in flower and it is easier to spot them among the vegetation. Later in the summer, even if the product’s quality is much better, it is more difficult to spot the orchids due to the surrounding vegetation or because the sheep graze the upper part of the plant.

Collectors uproot the plants with a shovel specially designed for salep, selecting the new tubers for collection. After collection, they wash and dry the tubers, using a needle and a thread, sewing them together and then hanging the string of tubers for drying if the quantity is small or they use large fabrics to dry the tubers in the sun for bigger quantities. Most of our informants claim to be unaware of the protection status of orchids and they think that the same rules are applied as for other medicinal plants. Nevertheless, the presence and controls of the local Police or Forestry Service in harvest areas, not only for orchids but also for other species of commercial interest (e.g. *Sideritis raeseri, Primula veris*) during summer 2018 is mentioned as a discouraging factor for commercial collectors. None said that they had observed a systematic decrease in natural populations, only large variability from year to year, which they attribute to variable winter and spring weather conditions, especially precipitation. Our informants consider wild boar disturbance of the habitat and bad collection practices, i.e. discarding the stems with the last-year tubers and not covering the dug after uprooting the plants, as the main threat to the species. On the contrary, good collection practices, i.e. replanting the remaining tuber and stem, are believed to help the plants spread their seeds, survive and flower again the following year.

### Demographic parameters and population profiles

All the demographic parameters observed in situ and calculated from field measurements are presented in Table 1. All sampling sites are characterized by presence of one- or two-leaf juveniles, although in the Anthochori site, due to the presence of dense grass, it was very difficult to spot them. The average number of flowers per shoot (FLN) varied among sites, with the highest observed in Lakmos and the lowest in Anilio (13.5 and 10.3, respectively). The number of fruits per shoot (FRN) on the other hand, was lowest in Lakmos and highest in Anilio (3.8 and 6.0, respectively). The percentage of pollination success differs among the sampling sites, ranging from 13.5% in Lakmos, up to 53.5% in Anthochori. Using the mean capsular seed number for *D. sambucina*, as proposed by Sonkoly et al. (as described in “Methods”), we found that in Anilio, the seed number per shoot (SNS) was larger than in all other populations (SNS= 18,084). Harvest status did not have any statistically significant influence on any of the demographic parameters we measured (*p* > 0.05 in all cases).

**Table 1:** Demographic parameters of the studied populations

Site code	N	JPR	IND	FLM	FRM	PS (%)	FLN	FRN	SNS
ANIL	119	Yes	30	310	72	23.2	10.3	6.0	18,084
ANTH	188	–^b^	50	634	215	33.9	12.7	4.3	12,960
TYMF	82	No	15	157	84	53.5	10.5	5.6	16,792
LAKM	151^a^	Yes	50	676	189	28.0	13.5	3.8	11,393

*N* population size, *JPR* presence of juveniles, *IND* number of individuals measured, *FLM* total number of counted flowers, *FRM* total number of counted fruits, *PS (%)* percentage of pollination success, *FLN* average number of flowers per stem, *FLN* average number of fruits per stem, *SNS* average seed number per shoot

^a^This population size for LAKM represents a cluster of a bigger population; the total size is estimated based on the area and field observations

^b^“–” = the presence of juveniles in ANTH could not be measured due to dense grass

### Disturbances and human presence on sites

All studied populations were impacted by human presence: in three out of four sites (except Anthochori), road proximity is high, and the populations are easily accessible. Human presence and activities were regular, with grazing (mainly sheep but also goats and cows) being the most common disturbance, except in Anthochori, which is turning from a regular and highly grazed area to an almost abandoned pasture, since only one shepherd uses the place for his herd. Tymfi was severely grazed, with numerous inflorescences missing from individuals, while extensive amounts of animal waste as well as human signs (discarded cigarettes and other mainly plastic litter) were also observed. In all sites *Helleborus odorus* subsp. *cyclophyllus* was present, a plant species that is linked to heavy grazing. Almost all sites hosted other plant species of collection interest, like mountain tea (*Sideritis raeseri* subsp. *raeseri*), common primrose (*Primula veris*) or the great yellow gentian (*Gentiana lutea*), that are all known to be excessively collected for medicinal use. In some populations like Tymfi and Lakmos we observed signs of pre-collection marking on *D. sambucina* individuals; at the first, we found plants that were excavated and then put back in place, while at the latter, we found plants excavated with their bulbs missing, as well as plants that were marked by leaving a circle of bare ground around them. Moreover, at two sites (Anilio–Lakmos), we observed big holes on the ground, footprints, as well as excavated and thrown plants with their tubers, evidence that point to the presence of wild boars in the area.

### Measuring populations and distribution

#### Individuals counted

The number of flowering individuals was counted in all four sites, Anthochori, Anilio, Tymfi and Lakmos respectively (Table 1). In the first three cases these were full local flowering populations, while the sample for Lakmos was part of a larger local population. Also, in Tymfi, only 15 out of the 82 total individuals actually carried flowers due to the heavy grazing in the area.

#### Scale-dependent density

The density measured in the four populations is different, ranging from 6.8 plants per hectare in the Anilio population to 218.8 in the Lakmos population. This is equivalent to a mean walking distance between nearest plants of 6.8 m in Lakmos to 38.4 m in Anilio. From Table 2, we see there is a high occupancy at larger scales for Anilio, with 43% occupancy at level-0; this means that in most replicate simulations, 7 out of 16 of the 128 m sub-squares are occupied.

**Table 2:** The density measured as the level of occupancy in the four populations we studied and the corresponding parameters for the fractal and Poisson models

Level	Width of cell (m)	Sub-cell width (m)	Total cells	Sampled/repl.	ANTH	ANIL	TYMF	LAKM
0	512	128	1	1	14%	43%	19%	14%
1	128	32	16	2	18%	20%	26%	13%
2	32	8	256	4	26%	13%	13%	23%
3	8	2	4096	8	12%	7%	8%	16%
4	2	0.5	65,536	16	23%	7%	7%	9%
r, Poisson rate/m^2^					0.00072	0.00045	0.00031	0.00058
Av. *p*_*F*_					0.19	0.18	0.15	0.15
*p* value					0.590	0.038	0.077	0.750

Also shown are the results of testing for an increase of occupancy with scale, that would indicate a Poisson pattern (uniformly spread throughout the landscape) by means of *p*-values

**Fig. 1:**
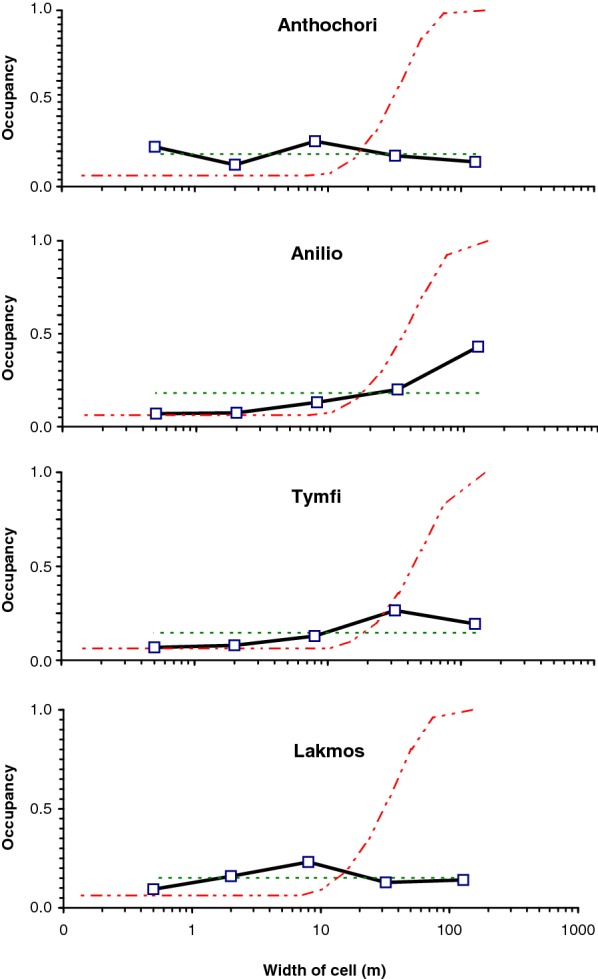
Patterns of population density as a function of cell size. Occupancy is the average proportion of sub-cells occupied when a cell is subdivided into 16 smaller units. In each case the width refers to the sub-cell size, not the mother-cell size. For each population the solid curve is the outcome of 1000 simulations obtained by locating the grid in slightly different positions relative to the population. The black line is the average. The thin broken line is the occupancy of the equivalent fractal model while the rising broken curve is the occupancy of the equivalent Poisson distribution

Although they do not entirely fit either a Poisson model (spatially-uniform) or fractal model (scale-free), the occupancy patterns in Fig. 1 are closer to fractal than to a Poisson distribution. Specifically, according to the *p*-values in Table 2, only in the case of Anilio does a regression test yield a significant indication of occupancy increasing with scale as might be expected with a Poisson. The fractal model dimensions fitted to the observed populations were all in the order of 1.26 to 1.38, which is a rather narrow band of values that may be fortuitous. The number of points we use is too small to provide strong evidence for a fractal model. It is however significant that there is evidence for a constant occupancy as a function of scale. The number of individuals in the Lakmos population is larger than 151 counted, as ours was only a sample within a small area. Under normal sampling assumptions (Poisson model), we assume that population scales with area (i.e. is proportional to *L*^2^). However, since the fractal model seems to be more consistent with our observations, the scaling regime is more likely to be fractional (i.e. in this case proportional to *L*^1.3^).

Table 3 summarizes some of the results of the population distribution survey. The area of the orchid meadows at Lakmos was calculated using QGIS version 3.6.0 ‘Noosa’ [44]. We observed the limits of the population in various directions in order to obtain a polygon containing the local population. We then found the area of the square enclosing this polygon. We calculated *L* as the square root of this area, then used Eqs. (2) and (3) to estimate the dimension and the total population, respectively. The salep yield was found using data from Sezik [18] that one kilogram of salep requires 2620 bulbs. Of the four populations, only Lakmos has a large number of plants and is capable of producing salep in substantive quantities.

**Table 3:** Population distribution features observed by us and their implications for salep production

Site code	Extent, *L*^2^ (ha)	Dimension *D* (est.)	Population *N* (est.)	Potential salep yield (kg)
ANTH	1.76	1.38	188	0.072
ANIL	17.58	1.30	119	0.045
TYMF	1.79	1.26	82	0.031
LAKM	1849.00	1.30	25,751	9.829

The estimate of the extent of Lakmos site was approximate, obtained by finding the extremes of the local population. The fractal dimension, *D*, was calculated using Eq. (2) and the population, *N*, using Eq. (3)

## Discussion

The Elder-flowered orchid, *Dactylorhiza sambucina* (L.) Soó, is one of the most important orchids collected for salep in Epirus. According to IUCN’s Red List Species assessment, the Elder-flowered orchid, although locally abundant across its range, it is considered to be in decline. This is argued on the basis of various threats, mostly linked with habitat change and human disturbances [45]. The species is also among those considered by Kreziou et al. [26], who note that orchid abundance has decreased in contrast to the past, and the present populations are likely to be severely affected by over-collection. So is this orchid threatened by current and anticipated levels of collection for salep? Like all orchids that grow in subalpine meadows, *D. sambucina* is subject to various threats and variability associated with its interactions with human presence, since this habitat is also used as pasture for livestock. Indeed, we observed grazing signs in all our study sites: absence of woody shrubs, low grass, species that are grazing indicators (e.g. *Helleborus odorus* subsp. *cyclophyllus*), and in some cases like Tymfi, we found numerous *Dactylorhiza sambucina* individuals with bitten stems. This is also noted in interviews; people who used to collect this species as salep, preferred to collect when the species was in flower, since it was easier to spot it, as later in summer the plants were eaten by grazing animals. When at high intensity and frequency, grazing can be an important factor that may increase the mortality, and thus, limit the overall reproductive capacity of populations in the meadows where the Elder-flowered orchids grow [46–48]. However, in Epirus, subalpine meadows have traditionally been used by transhumant shepherds as summer pastures [49, 50]; such grazing, which occurs during the reproduction period of the plant, seems to have had only small impact on the long-term viability of populations. However, when such pastures are left abandoned, then woody vegetation overgrows and affects orchid abundance [51], especially of species like *D. sambucina*, which need open spaces and grasslands to grow [52]. Indeed, intermediate levels of grazing can benefit orchids because it forestalls reforestation. Fischer and Wipf [53] found that in sites where grazing occurs, the percentage of shrubs and woody plants was low, while Köhler et al. [54] found that orchids can be benefited from moderate grazing, since it maintains low species competition, favourable conditions (space and lighting), as well as will enhance useful soil nutrients. The Mediterranean landscape is undoubtedly shaped by human presence [55], and its flora is replete with many examples of the co-evolution of plants with grazing animals [56, 57]. In comparison to the past, these meadows are now undergrazed [58]. It should be noted that, in Epirus, there is a current trend to introduce more cattle in sub-alpine pastures (personal observations), which replace sheep that traditionally graze summer pastures, causing the disturbance regime to shift from grazing to trampling. In local culture agreements and shepherds’ contracts, flock movements from lowlands to highlands (and vice versa) are marked by the holy days of the Orthodox military Saints, George (April 23rd) and Demetrius (October 26th) [59]. In addition to the concerns about reforestation or overgrazing, various other anthropogenic threats (e.g. wind farms and other human constructions), may negatively affect the habitat of *D. sambucina* in the future. However, at present we consider that this species is most likely to be impacted by collection for salep; a procedure accelerated by the rapid growth of the market for salep in recent years. It must be noted that, harvesting orchids for salep involves the removal of the tubers, which actually kills the plants, in contrast to grazing, that only removes the above ground parts (i.e. the reproductive tissues).

Despite protection of all Orchidaceae under European and Greek law, our study shows that orchids continue to be collected for salep in Epirus, and most respondents declared themselves unaware of the protection status of orchids. The growing popularity of salep can only be expected to increase pressure on populations in Epirus in the near future. The collectors in our study report a harvest of 10–100 kg per season. This can hardly be the lion’s share of imports into Turkey, as detailed by Sezik [18] (15–20 tonnes) and by recent reports of 80 tonnes mentioned by Hinsley et al. [60], neither of such export levels as described by Kasparek and Grimm [34]. Although the collection by locals in the areas we visited does not approach the pressures found elsewhere, our informants report increasing collection of salep by seasonal commercial collectors outside the local community. The most important question is to what extent traditional and modern harvesting pressures affect population sizes and their distributions. According to Kreziou et al. [26] “*The annual harvests of thousands of 50* *kg units of salep reported in the 1850s … no longer occur, as orchids have become rare and remaining populations have lost resilience to such harvesting pressure*”. Ultimately, estimates of population resilience need to be associated with responses to measurable pressures. Thus, a survey of population performance under a harvesting regime is needed. Our basic survey is an important step towards a more integrated perspective for salep orchids. In fact, our study could not find empirical support for statistically significant differences between harvested and non-harvested study sites in any demographic or population parameter. At our four sites, distributions of populations are quite similar, exhibiting similar multi-scale patterns of occupancy. When we compare a model for a uniform distribution (Poisson) with a fractal model only one site shows a pattern of occupancy significantly more similar to uniformity. For example, neither Lakmos, the most harvested, nor Anthochori the non-harvested population, were different. Of course, the small size and span of our data (four sites of size ~ 1 ha for 1 year) limits the scope of conclusions we can draw from these results, and so they should be regarded as informative rather than conclusive. The low numbers of orchids in three of the sites (insignificant for salep collection) and high number in Lakmos are consistent with large spatio-temporal variability, as reported by our informants, and this could be calibrated better in a long-term study. Thus, our study underlines the need for longer-term observations of the response of orchid populations to harvesting. Some of the areas we observed have been heavily harvested for generations. So, why are there still signs of abundance in these areas, such as continuing collection for salep that requires many plants to be removed? Should salep orchids be rarer or more common? Salep is reported as collected for generations in a locally-based traditional way and it is common to be sold in herbal markets [21], but on occasion it has been fashionable across Europe [8]. The collection history of salep in Epirus is not stable, but has major fluctuations, depending on market demand. For example, salep was a very popular beverage in the eighteenth century [31] but more recently, in the 1960s, it was in high-demand from pastry shops in Athens, where it was used for the production of the traditional Greek ice-cream “kaimaki” as mentioned in our interviews with older collectors. There followed a reduction that affected mainly the street salep vendors and recently salep is in the spotlight again, due to the revival of natural products worldwide and also in Europe [36]. The consumption behaviour is strongly influenced by geographical location and neighboring countries. Not only tradition but external input such as trade and cultural exchange are strong factors shaping consumption behaviour (like in mushrooms) [61]. Perhaps our sense of abundance in Site-4 is an illusion caused by the “shifting baseline” [62]. Populations a few 100 years ago could have been much larger. Such information might be obtainable through longer-term historical and archive studies on the production and consumption of salep in Epirus. We are thus drawn to the question of what level of harvesting is likely to cause local extinction. The sustainability of harvest is related to the reproduction strategies of specific plants, and in the harvesting practices associated with them. For example, management of medicinal plants can be improved by taking harvesting patterns, plant life forms and growth patterns into consideration [63]. However, our results do not show strong sensitivity to harvesting pressures in population demographic parameters and distributions. So, there may not be a simple connection between harvest intensity and extinction risk in these types of organisms. Orchids are characterized by very high fecundities and high seed mortality, leading to very high population variability [64] and have a life cycle that is characterized by periods of dormancy or vegetative states, so our observations may be missing important sections of the populations. Concepts like maximum sustainable yield may be harder to identify for these kinds of organisms than for mammals or birds. Counterintuitive patterns of insensitivity to harvest levels that have been widely observed for mushrooms [65] or sea-turtles [66], may be linked to this high-fecundity life-strategy [64]. Elsewhere it has been argued that range size is one of the best predictors of risk of local extinction from habitat loss [67, 68], so that range rather than collection pressure determine its resistance to anthropogenic pressures such as collection. Overall, it remains an important challenge to establish conservation boundaries for these orchid populations.

Positive developments include rapid developments in artificial germination and cultivation of orchids [69–71] and in situ and ex situ conservation efforts [72]. For this kind of species, that are highly demanded in the current market, proper management of their wild populations, ex situ conservation, as well as clonal propagation methods should be highly prioritized in the conservation agenda. Of course, there are still worries that a taste for “real” salep might activate an “anthropogenic Allee Effect”. That is that people place disproportionate value on rare species [73], valuing the wild-harvested article over the cultivated one and generate a cycle of increased exploitation and rarity ultimately leading to its extinction in the wild [74].

## Conclusions

Our results show that traditional style salep collection continues in Epirus, and that modern commercial collection for a growing mass-market may already be underway. This added collection is expected to put orchid populations under increased pressure. However, at present, local collectors do not report problems of abundance. In addition, our own pilot study of the demographics and distribution of *D. sambucina* populations in the area did not find strong indications of differences between harvested and non-harvested sites for any parameter. This study underlines the need for wider and longer-term observations of wild orchid populations. Also needed is a better model of how wild orchid populations respond to harvesting at different intensities.

## Methods

### Site selection

The northern part of Pindos Mountain Range, a region with high geomorphological heterogeneity (e.g., limestone rocks, screes, ultramafic outcrops) constitutes our wider study area. It also has a wide variety of habitats (e.g., Bosnian and black pine, fir, beech and oak forests, grasslands with *Nardus stricta*, oro-mediterranean heathlands and ravine forests) [28]. It hosts ca. 30% of the total orchid species present in Greece [75, 76], and is especially rich in dactylorchids (e.g. *Dactylorhiza sambucina*) [28, 29], some of the main salep orchids in Greece. Northern Pindos has also been identified as a place under, or soon to be under, higher collection pressure for salep [26, 77]. For our study we selected four (4) sampling sites: Anilio (ANIL), Anthochori (ANTHO), Tymfi (TYMF) and Lakmos (LAKM). Anilio, Anthochori and Tymfi were identified as frequent collection areas according to local collectors, while Anthochori site, although mentioned as a salep meadow during the interviews, none of the participants indicated the specific place as a harvesting site. According to this, the selected sites were divided into two separate categories, depending on their harvest status (collected/not collected) (Table 4). All sampling sites belong to the Pindos geotectonic zone and more specifically, three of them occur on limestones (Anilio, Anthochori and Lakmos) and one on flysch (Tymfi) [78]. The sites do not differ much in terms of pH (6.0–6.2) or altitude. The slope varies a little bit more (12–27%).

**Table 4:** Description of sampling sites

Site	Code	Habitat type	Harvest status	Altitude (m) and slope	Co-occurring orchid species	Other species of collection interest
Anilio	ANIL	Grassland—Beech forest opening	Collected	1460 26%	*Orchis mascula*	*Satureja montana*
					*Neotinea ustulata*	*Primula veris*
					*Anacamptis morio*	
Anthochori	ANTHO	Subalpine meadow	Not collected	1541 27%	–	*Gentiana lutea*
						*Primula veris*
						*Sideritis raeseri*
Tymfi	TYMF	Subalpine meadow	Collected	*1553* *12%*	*Platanthera chlorantha*	*Thymus* sp.
					*Neotinea ustulata*	*Acinos* sp.
					*Anacamptis morio*	*Hypericum* sp.
Lakmos	LAKM	Subalpine meadow	Collected	1630 16%	*Orchis mascula*	*Gentiana lutea*
					*Neottia ovata*	*Primula veris*

### Study species

**Fig. 2:**
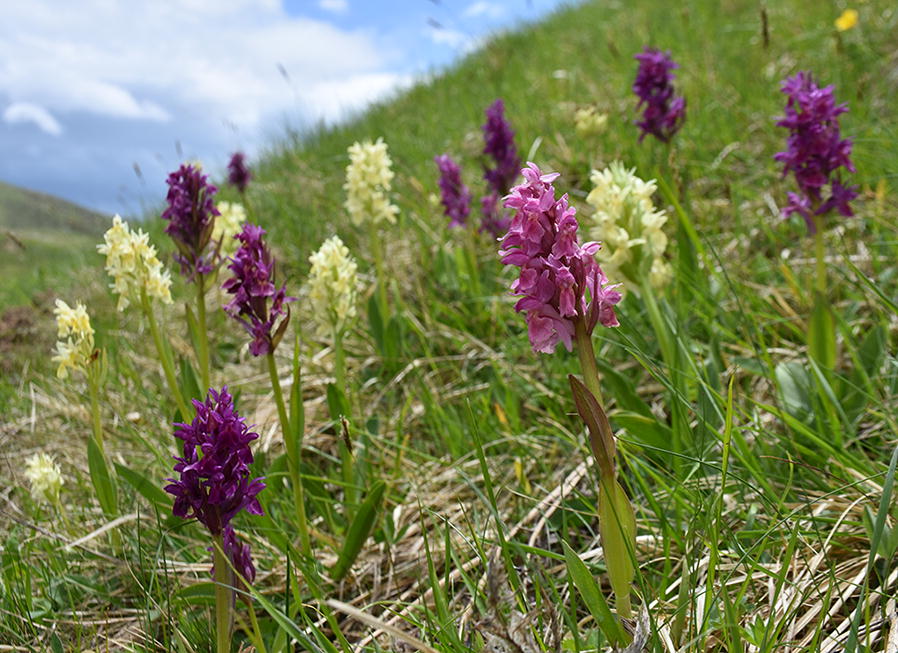
The Elder-flowered orchid, *Dactylorhiza sambucina* (L.) Soó (1962), showing its characteristic color variation (photo credits: Kalliopi Stara)

We selected *Dactylorhiza sambucina* (L.) Soó (1962) as our study species, which is commonly known as the Elder-flowered orchid and constitutes a European species, with a distribution ranging from Scandinavia down to Sicily, and from central Spain to Crimea in the East. At its southern borders it can be found in mountainous areas, in a variety of habitats including sub-alpine and alpine meadows, forest edges, open grasslands and woodlands. It usually occurs in fully sunlit places, on alkaline to slightly acidic damp soil, at altitudes of up to 2600 m a.s.l. [79]. In Greece, it is relatively common in the country’s north and central mountainous areas (mainly across the Pindos range), where it can form large colonies, mostly in sub-alpine meadows. A distinctive feature of this orchid is its flowers’ colour, ranging from whitish-yellow to light or dark violet (Fig. 2). An intermediate form with pink flowers is also sometimes observed (var. *zimmermanii*) [80]. *Dactylorhiza sambucina* is a food-deceptive orchid, pollinated mainly by bumblebees, with a flowering period between (late April-) May and July, depending on altitude [52, 80]. According to IUCN, the current extinction risk of *D. sambucina* is low (a “Least Concern” taxon), but the species’ population trend is thought to be decreasing, mainly due to habitat loss and other anthropogenic threats [45].

### Interviews

In order to investigate the relation of local inhabitants of Epirus with orchids, we carried out interviews with *key informants* and other stakeholders in villages near the areas for which we had prior indication for collection ([77], and personal observations prior to 2018). We use the term “*key informants”* to define people with knowledge that is more extensive, detailed or privileged than ordinary people, and thus, particularly relevant for this research [81]. In this research, key informants were expected to be collectors for private or commercial use. We approached informants using the *snowball sampling method*, where research participants suggest other familiars (friends, relatives, neighbors etc.) to participate in the study [82]. Interviews were carried out in five (5) villages that are the nearest to the selected sites (Anthochori, Krania, Milia, Anilio, Vradeto) and in Ioannina town. They began with a photo exercise based on colour photographs of the species *Dactylorhiza sambucina, Orchis mascula* and *Ophrys helenae* (not characterized as a salep orchid, but is found in the area). We asked participants to name the species and comment on abundance, characteristics that differentiate species and local names. We also asked about the use of orchids for salep (reasons of use, preparation of the beverage), the collection (areas and time of collection, how many years they collect, by whom they learned the practice, special tools, quantities). Other questions were about abundance, prices, qualities, market demands and processing after collection. There were also questions concerning nature conservation: past/present differences in the biotope, species abundance and perceived trends in natural populations, threats, conservation status of the species, protection measurements, practices that could ensures species survival and sustainable management. Interviews with local informants (60 in different villages across Pindos) took place between May 2018 and September 2019. There are also additional interviews conducted by team members prior to the project [77]. Herein, we analyze results from 15 interviews directly related with the areas where we implemented measurements in the field. We conducted the interviews in private houses (2), cafes or tavernas (6), in the field (5) and in shops that sell salep (2). Each interview lasted from 10 to 90 min and in one case it was followed by a joint visit with two local informants and three researchers to the field. In all, we interviewed 15 informants (11 men and 4 women), aged 40–74 years (10 people) or younger than 40 (5 people) (Table 5). Six of them were characterized as key informants. Information was documented by written notes and was supplemented by video or voice recording, with the permission of the informants.

**Table 5:** Results from interviews conducted at the nearest villages to the selected sampling sites

Site	Informants				Uses of *D. sambucina*			
	Age	Μen	Women	Total	Commercial	Private	None	Unknown
TYMF	< 40	1		1	1			
	40–74	3		3	2		1	
ANTH	< 40	1	1	2				2
	40–74	2	1	3				3
ANIL	< 40	2		2	2			
	40–74	1	1	2			2	
LAKM	< 40							
	40–74	1	1*	2	1*	1		
Totals		11	4	15	6	1	3	5

Each line represents answers by one demographic category and how they use *D. sambucina*. The asterisk (*) refers to a woman who collected commercially in the past, but not currently. Note that “None” in column 8 means that the informants could identify the species but they do not collect it

### Orchid fecundity, recruitment, and population profiles

For our field study, we created a protocol divided in two sections, in order to apply to all of our sampling sites. The first section of the protocol included all the parameters that describe each sampling site. Natural characteristics of the site (e.g. altitude, slope, habitat type, co-occurring orchid species), land-use information and indications of harvesting (e.g. distance from road or other infrastructure or settlements, grazing, markings, signals, species of collection interest etc.), as well as observations in situ (e.g. remaining plant part, holes etc.) were included (modified field protocol of Molnár et al. [83]). The second section of the protocol focused on demographic parameters. In this section, we included parameters dealing with the above-ground part of the species’ life cycle, which are directly linked to fecundity and recruitment, and can possibly be affected by salep harvesting. Thus, in each sampling site, we counted the number of flowering individuals (*N*), and we marked the presence or absence of seedlings (juveniles—JPR). According to the population size, the flowering stage, and the overall demographic status of each population, we selected 30 to 50 individuals, and counted the total number of flowers (FLM) and fruits per stem (FRM) in each individual. The only exception was the Tymfi population, where only 15 out of the 82 total individuals were deemed suitable for measurements (fully developed inflorescence), due to severe grazing in the area. From our field measurements, we estimated the average number of fruits and flowers per shoot (FLN and FRN respectively) for non-grazed plants, as well as the percentage of pollination success (PS%), defined as the ratio of number of fruits divided by the number of flowers. Using the mean capsular seed number for *Dactylorhiza sambucina* proposed by Sonkoly et al. [84], we calculated the seed number per shoot (SNS). Finally, we tested whether there any differences among these parameters measured, caused by harvest status via a one-way analysis of variance (ANOVA), or where heteroscedasticity was high, with Kruskal–Wallis tests.

### Measuring populations and distribution

For the four sampling sites surveyed, we estimated the population size, based on the number of flowering individuals. For each population we noted the coordinates of all observed flowering individuals of *D. sambucina* within a square. For three of the four populations, this was a full count of the local population (those in flower). For the fourth (Lakmos), the cluster was a sample of the larger population. In describing plant distributions, it has been widely recognized that a multiscale approach is helpful [85–87]. Thus, we estimated the population density and extent on five different spatial scales. We examined the same distribution on different spatial scales by looking at the grid-occupancy on nested grids of different cell sizes. The distribution of a population can be understood by how it occupies area at different scales. If the distribution is like Poisson, the proportion of occupied cells should increase as the size of the grid cell increases. If the distribution is more towards fractal, the proportion of occupied cells is much the same regardless of spatial scale.

The inclination of the landscape means that the squares in our analysis are not the same size as the area of squares on the ground. Also, since the slope is changing in places, squares are not all the same size in different sites or at different points within the same site. Nevertheless, this error is not so great in our case because most of the slopes are about one in four (one metre rise for 4 m horizontal), which leads, by Pythagoras’ Theorem, to an increase in size of sqrt(1 + 16) ~ 4.12, that is 3.1% increase in area. In ecological systems this error is fairly small and so we do not think it causes a major problem.

### Estimating scale-dependent density

**Fig. 3:**
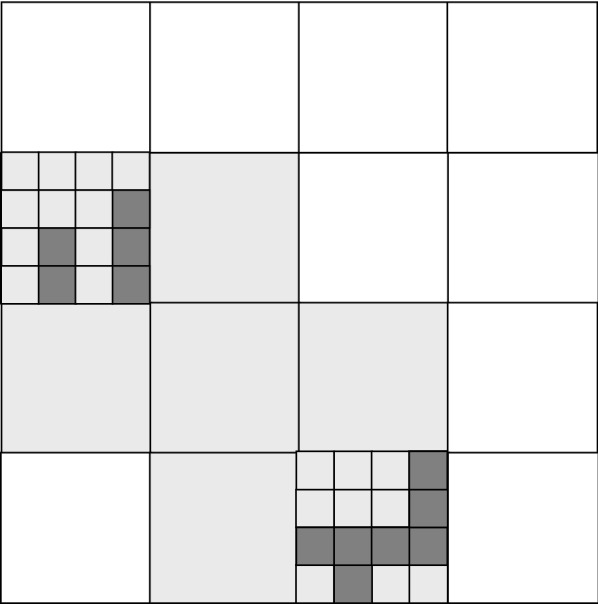
Basic geometry of grid on multiple scales

In this approach, we estimated the grid occupancy on different spatial scales by using a grid with successively finer cell sizes. At a given scale, the proportion of cells occupied tells us whether the distribution is *even* at that scale (many occupied cells) or *clustered* at that scale. We move to a finer scale by dividing each occupied square into 16 sub-squares (Fig. 3). For each population studied, we initially defined a square of size 512 m with major axis aligned North–South around it. The size chosen for the bounding square of 512 m is so that all populations will be analyzed with the same scale and for convenience so that we have five different scales starting with a square of side 2 m. In several cases (such as in Anilio), this area included features such as forest and roads where the plant cannot grow. Also, failure to occupy the square may be due to these but also to unseen factors such as orchid mycorrhizae. This is denoted level-0. At this level there was a single occupied square, so the average occupancy (proportion occupied) at this scale *L* is *p*_0_ = 1. The initial square was divided into 16 (“level-1”) sub-squares of size 128 m and we counted the number of these occupied, *n*_1_. The average occupancy (proportion occupied) at the scale 128 m was thus *p*_1_ = *n*_1_/16. In general, *p*_*k*_ denotes a proportion of sub-squares, of size 512/4 ^*k*^ in width, that are occupied, within a mother square of size 512/4 ^*k*−1^. We selected two of the occupied level-1 squares at random. If only one was occupied (n_1_ < 2) we selected this occupied square. For each selected square, we proceeded to the next level, repeating steps 1 and 2 above. The occupancy was the average value obtained over all selected squares at this level. We repeated the above steps for the next levels until we reached the level where sub-squares are on the order of plant size, namely a width of 0.5 m. We noted the occupancies *p*_0_–*p*_4_ at the five different scales of square (cell) size 512, 128, 32, 8 and 2 m. We repeated steps 1 to 5 for 1000 replicate simulations. Each time the initial square enclosing the population was displaced slightly at random, constrained only to contain all the observed points. The average occupancies over the many replicates offers an estimate of the density of plants at four different scales: $\bar{p}_{1} ,\bar{p}_{2} ,\bar{p}_{3} \;{\text{and}}\;\bar{p}_{4}$.

### Poisson model

We fitted a Poisson model to the data. In the Poisson model of spatial distribution, each unit of area has a fixed probability of occupation. The Poisson model is a basic assumption in most non-stratified sampling schemes. If we find *N* individuals in an area of size *A*, then we fit a Poisson model by finding *r*, the Poisson process rate, estimated by *N/A*. The probability that an area *a* is empty is given by *p*_0_ = *e*^−*rA*^. Thus as we move to larger scales (so area *A* gets bigger by 16-fold each time) the probability of a cell being empty falls rapidly. Thus, if a population is distributed randomly according to the Poisson model there is a dramatic difference in occupancy between scales, with larger areas having much higher probabilities of being occupied than smaller ones. In the Poisson model, there is a characteristic spatial scale, which is sometimes called the *mean free path*. This is the shortest distance an observer will have to walk between a single plant and the next one. Any randomly chosen area much smaller than this scale will probably be empty but areas much larger than this scale will be overwhelmingly occupied. In a grid-based system, for a Poisson model, as cell-size increases so does the probability of occupancy. Thus, average occupancy should increase with scale. To predict the occupancy of a given cell of area *a* in this model, we use the fact that the probability of occupation of a single cell is 1−exp(−*ra*), so that the expected number of occupied out of 16 cells is 16(1−*e*^−*ra*^).

### Fractal model

We also fitted a fractal model to the data. A fractal model of distribution has equal occupancy at all scales. If a population is distributed spatially according to a fractal model there is no systematic variation of occupancy between scales and there is no characteristic spatial scale. Fitting a distribution to a fractal model means finding best-fit fractal (usually from a specific type of fractal) to the data. An important class of fractals consists of the so-called *percolation fractals* [88]. These are constructed by iteratively subdividing an occupied area into equally-sized sub-areas and defining each sub-area as occupied or empty according to a fixed probability generates such a distribution [89]. The probability (*p*_*F*_) and the degree of subdivision determines the *fractal dimension*:

1 $$D = \frac{{\log (m^{2} p_{F} )}}{\log (m)}.$$

Here *m* is the number of divisions at each level (in our case 16) and *p*_*F*_ is the proportion of occupied sub-squares at each level. We can assume a percolation fractal model for the distribution observed and then fit this equation using the average of the occupancies $\{ \bar{p}_{1} ,\bar{p}_{2} , \ldots ,\bar{p}_{K} \}$ for *p*_*F*_. Thus if we find the average value of Eq. (1) for *K* different scales, each with a different $\bar{p}_{j}$, we have:

2 $$\hat{D} = 2 + \frac{\log \left\langle p \right\rangle }{\log (m)}.$$

Here <*p*> is the geometric average of $\{ \bar{p}_{1} ,\bar{p}_{2} , \ldots ,\bar{p}_{K} \}$ and as $\left\langle p \right\rangle < 1$, the value of $\hat{D}$ is always less than 2. If we assume this fractal model, we can find the fractal dimension using Eq. (2). This can then be used to estimate the population [90]. For fractal distributions, the population inside a square of side *L* increases according to the power law, *N* = *BL*^*D*^, and then we can extrapolate population from a sample to a larger area, we can use the approximate formula:

3 $$N_{2} \approx \left( {\frac{{L_{2} }}{{L_{1} }}} \right)^{{\hat{D}}} N(L_{1} ).$$

In this approach we estimate the grid occupancy on different spatial scales by using a grid. Thus, the important parameter here, in the context of our ecological system, is the occupancy probability, *p*_*F*_, which in the fractal model of this kind, is considered a constant. Thus fractal model can be fitted by finding the average occupancy over different scales.

### Distinguishing a poisson from a fractal pattern

From an ecological perspective, we are interested in deciding whether these orchids are more uniformly spread throughout the site (Poisson model) or whether it corresponds to uniformity in scale (fractal model). To distinguish a fractal model from a Poisson model, note that in the Poisson model the occupancy will increase with scale whereas in the fractal model it remains constant with scale. To decide whether the observed spatial patterns were more consistent with Poisson or Fractal patterns we find the occupancy of a square by its 16 sub-squares at scales 512 m, 128 m, 32 m, 8 m and 2 m by the 16 sub-squares of side 128 m, 32 m, 8 m, 2 m and 0.5 m, respectively, to give *p*_*F*_ at different scales. We then tested the slope of the regression of *p*_*F*_ against log *L*.

## Data Availability

The datasets in this paper are available from the corresponding author on reasonable request.
